# Identification of Lethal Inhibitors and Inhibitor Combinations for Mono-Driver versus Multi-Driver Triple-Negative Breast Cancer Cells

**DOI:** 10.3390/cancers14164027

**Published:** 2022-08-20

**Authors:** Geng Chia Ku, Abygail G. Chapdelaine, Marina K. Ayrapetov, Gongqin Sun

**Affiliations:** Department of Cell and Molecular Biology, University of Rhode Island, 120 Flagg Rd, Kingston, RI 02881, USA

**Keywords:** triple-negative breast cancer, DU-4475, MDA-MB-231, BRAF V600E mutation, Src kinase, KRAS, combination targeted therapy, pharmacological synthetic lethality, mono-driver cancer, multi-driver cancer

## Abstract

**Simple Summary:**

Triple-negative breast cancers lack estrogen and progesterone receptors and HER2. They lack effective targeted therapies and tend to be more aggressive, with the worst five-year overall survival and a higher rate of recurrence. This study investigates the oncogenic signaling mechanisms of two model cell lines, DU-4475 and MDA-MB-231. The former is a mono-driver cancer cell line relying on a BRAF V600E mutation and the latter is a multi-driver cancer dependent on a KRAS-mutation-activated MAP kinase pathway and Src kinase. The results of this study reveal that while the mono-driver cancer cells can be effectively killed by one drug blocking their predominant driver, multi-driver cancer cells can only be killed by drug combinations blocking all drivers. The drug combination in MDA-MB-231 achieves strong synergy and potent synthetic lethality. This study suggests pharmacological synthetic lethality as a foundation for combination targeted therapy for multi-driver cancers.

**Abstract:**

There are no signaling-based targeted therapies for triple-negative breast cancer. The development of targeted cancer therapy relies on identifying oncogenic signaling drivers, understanding their contributions to oncogenesis and developing inhibitors to block such drivers. In this study, we determine that DU-4475 is a mono-driver cancer cell line relying on BRAF and the mitogen-activated protein kinase pathway for viability and proliferation. It is fully and lethally inhibited by BRAF or Mek inhibitors at low nM concentrations, but it is resistant to inhibitors targeting other signaling pathways. The inhibitory lethality caused by blocking Mek or BRAF is through apoptosis. In contrast, MDA-MB-231 is a multi-driver triple-negative breast cancer cell line dependent on both Src and the KRAS-activated mitogen-activated kinase pathway for proliferation and viability. Blocking each pathway alone only partially inhibits cell proliferation without killing them, but the combination of dasatinib, an Src inhibitor, and trametinib, a Mek inhibitor, achieves synthetic lethality. The combination is highly potent, with an IC_50_ of 8.2 nM each, and strikingly synergistic, with a combination index of less than 0.003 for 70% inhibition. The synthetic lethality of the drug combination is achieved by apoptosis. These results reveal a crucial difference between mono-driver and multi-driver cancer cells and suggest that pharmacological synthetic lethality may provide a basis for effectively inhibiting multi-driver cancers.

## 1. Introduction

Breast cancer is the most common cancer in women and metastatic breast cancer is the second-leading cause of cancer-related deaths in American women [1,2]. Triple-negative breast cancer (TNBC) lacks an estrogen receptor (ER), progesterone receptor (PR), and HER2 receptor. It accounts for ~15% of all breast cancers [3,4,5] and tends to be more aggressive [6,7,8], with higher mean tumor size, tumor grade, the worst five-year overall survival, and a higher rate of recurrence [9,10,11]. It is more common in premenopausal and African-American women [12,13,14,15].

A major reason for the poor outcomes for TNBC patients is the lack of effective targeted therapies. Hormonal therapy, targeting ER [16,17], and small-molecule and antibody therapies, targeting ErbB2 [11,18,19], have significantly improved treatments for ER^+^ and HER2^+^ breast cancer patients. TNBC lacks these receptors and is heterogeneous in its oncogenic mechanisms without predominant targets. Consequently, advanced TNBC still relies on chemotherapies with anthracyclines, alkylating agents, and taxanes [20,21,22]. Metastatic TNBC has a 5-year survival rate of only 12% [23,24]. The lack of targeted therapy for TNBC also epitomizes a general problem for targeted cancer therapy. In 2018, only 8.33% of all US cancer patients were genomically eligible for targeted therapies and only 4.9% benefited from such treatments [25]. Establishing predictive relationships between biomarkers and effective treatments is urgently needed for better treatment of TNBC and many other cancer types.

Among the eight cancer hallmarks [26,27], at least six are fully or partially supported by oncogenic signaling: sustaining proliferative signaling, evading growth suppressors, resisting cell death, inducing angiogenesis, activating invasion and metastasis, and reprogramming energy metabolism. Thus, blocking cancer signaling drivers is expected to be a key component of effective targeted cancer therapies for TNBC [28,29,30,31,32,33]. TNBC frequently displays an activated phosphatidylinositol 3-phosphate (PI3K) pathway due to PIK3CA mutation (9%), PIK3CA amplification (49%) [3], PTEN mutation/loss (35%), and INPP4B loss (30%). Many components of the mitogen-activated protein kinase (MAPK) pathways are also amplified in TNBC, including KRAS (32%) and BRAF (30%) [3]. Some receptor protein tyrosine kinases (rPTK), including EGFR, FGFR1, FGFR2, IGF-1R, KIT, MET, and PDGFRA, are also amplified in TNBC [3]. Targeting the PI3K pathway (PI3K [34,35,36,37] and AKT [38,39,40]), MAPK pathway [41,42,43,44,45], or PTKs (EGFR [46], FGFR [47,48,49], and Src kinase [50,51,52]) has not been effective in treating TNBC in clinical trials.

While the underlying reasons for the lack of success in these clinical trials may be complex, one plausible explanation is that most TNBC tumors are supported by multiple oncogenic drivers, making targeted therapy against any one driver not effective. This possibility can be fully explored by determining the oncogenic mechanisms in TNBC tumors and model cell lines, while this obstacle can be overcome by developing effective drug combinations against multi-driver cancers. This study demonstrates that TNBC cell line DU-4475 is a mono-driver TNBC model predominantly driven by a BRAF V600E mutation. We then compare the pharmacological properties of DU-4475 to those of MDA-MB-231, a multi-driver TNBC cell line. The results reveal striking differences between mono-driver and multi-driver cancer cells in their responses to protein kinase inhibitors and suggest pharmacological synthetic lethality may provide a mechanistic basis for treating multi-driver cancers.

## 2. Materials and Methods

### 2.1. Cell Lines, Media, and Drugs

The human TNBC cell lines, DU-4475 and MDA-MB-231 were purchased from ATCC (Manassas, VA, USA). ATCC authenticated them by short tandem repeat profiling, cell morphology monitoring, karyotyping, and cytochrome C oxidase I testing. The cells were grown in RPMI-1640 and DMEM media, respectively, with 10% FBS and 1% penicillin-streptomycin (Thermo Fisher Scientific, Pittsburgh, PA, USA). They were cultured at 37 °C in humid atmosphere containing 5% CO_2_. Kinase inhibitors were purchased from Selleckchem (Munich, Germany), LC Laboratories (Woburn, MA, USA), MedChemExpress (Monmouth Junction, NJ, USA), or AdooQ Bioscience (Irvine, CA, USA).

### 2.2. Cell Culture and Viability Assays

Cell culture and viability assays were performed as described previously [53]. The effect of a drug on cell viability was determined in 96-well plates using Biolog Redox Dye MA or MTT dye assay (Thermo Fisher Scientific) per the manufacturer’s instructions. DU-4475 cells were cultured in suspension and plated at 10,000 cells per well in a 100 µL medium containing the indicated drug and 1% DMSO. The cells were treated with indicated drugs for 48 h at 37 °C with 5% CO_2-_ before the cell viability was determined using the Biolog Redox Dye MA by calculating the differences in absorbance at 590 and 750 nm. MDA-MB-231 cells are adherent cells and were seeded at 12,000 cells per well in 120 µL and allowed to attach overnight. A culture medium containing the drug and 5% DMSO (30 µL) was added to each well and the cells were cultured for 48 h before the cell viability was determined by the MTT assay. The formazon product was determined by absorbance at 490 and 750 nm using a Biotek Microplate Reader. In the MTT assay, the A_490_–A_750_ values were taken as indicators of cell viability. All cell growth and drug inhibition experiments were performed at least twice in triplicate.

### 2.3. Curve Fitting by the Hill Equation and the Biphasic Equation

Dose–response data were fitted to the Hill equation and the biphasic equation in Microsoft Excel using the Solver add-in program. The Hill equation used was I = I_max_ × D^n^/(IC_50_*^n^ + D^n^), where I_max_ is the maximal inhibition by a drug, IC_50_* is the IC_50_ for inhibiting the portion of cell viability that is sensitive to the drug, and is the Hill coefficient or slope. The biphasic equation used was I = F_1_ × [D]/([D] + K_d1_) + F_2_ × [D]/([D] + K_d2_), where the inhibition of cell viability (I) as a function of variable drug concentration is determined by three constants: F_1_, K_d1_, and K_d2_. The F_2_ was not an independent variable but instead calculated as 100%-F_1_. In Hill equation fitting, the root mean square error (RMSE) was minimized using the I_max_, IC_50_* and *n* as variables. In biphasic curve fitting, the RMSE was minimized using the F_1_, K_d1_, and K_d2_ as variables.

### 2.4. Drug Synergy Analysis and Combination Index Calculation

Drug synergy was evaluated by the combination index (CI) and the dose reduction index (DRI), as described previously [53,54]. Cell viability was determined after incubation in the presence of each drug alone or both drugs (1:1 ratio) at 16 concentrations ranging from 0.6 nM to 20 μM. The CI was calculated according to Chou [55], using the Chou and Talalay Method [55] with the following equation: CI = IC_x-AB_/IC_x-A_ + IC_x-AB_/IC_x-B_, where IC_x-A_, IC_x-B_, and IC_x-AB_ are the concentrations of drug A, drug B, and drug AB combination, causing X% inhibition of cell viability, respectively. DRI was calculated as 1/CI.

### 2.5. Time Course Experiments

To determine the time-dependent effects of drug treatments on cell viability of DU-4475 and MDA-MB-231, cells were treated with drugs at indicated concentrations as described in Section 2.2 and the cell viability was determined 1 h, 24 h, 48 h, and 72 h after the treatments were initiated.

### 2.6. Cell Treatments and Western Blots

To determine the effects of a protein kinase inhibitor or a combination of inhibitors on the signaling proteins, cells were seeded at 70% confluency and treated with drugs at indicated concentrations for 1 h under normal cell culture conditions. After the treatment, the cells were placed on ice, washed 1× with chilled PBS, and lysed in RIPA buffer containing a protease inhibitor cocktail (Sigma-Aldrich, St. Louis, MO, USA) and protein phosphatase inhibitors (PhosSTOP, Sigma-Aldrich) for 30 min at 4 °C. Lysates were cleared at 21,000× *g* for 10 min at 4 °C. Protein concentrations of the supernatants were then quantified using a BioRad Bradford Protein Assay. Then, 12–40 μg of protein was separated using a gradient (4–20%) SDS-PAGE gel and transferred onto a nitrocellulose membrane. The membranes were then blocked in 5% non-fat milk in TBST (Tris-buffered Saline with Tween 20) for 1 h before probing with the indicated primary antibody overnight. Membranes were imaged using LI-COR Odyssey CxL Imager and analyzed using Image Studio software (LI-COR Biosciences, Lincoln, NE, USA). All antibodies were purchased from Cell Signaling Technology (Danvers, MA, USA). The density of each protein band in the Western blots was measured using ImageJ 1.53K software [56]. 

### 2.7. DU-4475 Apoptosis and Necrosis Assay

DU-4475 cell apoptosis and necrosis in response to trametinib treatment are monitored using the RealTime-Glo Anexin V Apoptosis and Necrosis assay (Promega, Madison, WI, USA) [57]. DU-4475 cells were seeded at 10,000 cells per well in 100 μL medium with or without trametinib in 96-well white plates with clear bottoms. Apoptosis and necrosis assay reagents (100 μL) were added and the cells were incubated at 37 °C and 5% CO_2_. Each well contained 1% DMSO, 20 nM trametinib (treatment) or no drug (control). The plate was shaken for 30 s at 500–700 rpm before the luminescence and fluorescence (485 nm_Ex_/528 nm_Em_) in each well were recorded every 6 h, using a Biotek Synergy HTX multi-mode reader (Agilent Technologies, Santa Clara, CA, USA). The assay was performed in quadruplicates and the average ΔLum (luminescence of treatment—control) and ΔFluo (fluorescence of treatment—control) are reported.

## 3. Results

### 3.1. DU-4475 Cell Line Is Exceptionally Sensitive to BRAF and Mek Kinase Inhibitors

We previously reported that TNBC cell lines, MDA-MB-231 and MDA-MB-468, are each dependent on two signaling pathways and can be effectively inhibited by inhibitor combinations blocking both pathways [53]. To evaluate if most or all TNBC cell lines are multi-driver cancer cells that cannot be blocked by any individual signaling kinase inhibitors, we examined the drug response data of all TNBC cell lines in the Genomics of Drug Sensitivity in Cancer database [58]. Table 1 lists 17 TNBC cell lines and the IC_50_ for each cell line’s most potent protein kinase inhibitors for targeted therapy. Most of the TNBC cell lines do not respond potently to any protein kinase inhibitors, displaying high nM to low μM IC_50_s. The only exception is DU-4475, which is potently inhibited by the BRAF inhibitor dabrafenib (IC_50_ = 6.3 nM) and the Mek inhibitor trametinib (IC_50_ = 0.5 nM). These data suggest that most TNBC cell lines do not depend on any single kinase for viability, while DU-4475 is dependent on BRAF and Mek for viability. This suggestion is consistent with DU-4475 being a mono-driver cancer cell line and the others reported are multi-driver cancer cell lines.

In this study, we aimed to determine whether DU-4475 was truly a mono-driver cancer cell line. DU4475 was derived from a cutaneous metastatic nodule from a patient with advanced TNBC [59,60]. Even though DU-4475 has been widely used as a model cell line to study the oncogenesis of TNBC, the oncogenic mechanism of this cell line is not established. According to the COSMIC database [61], DU-4475 contains two notable mutations: a truncation mutation at E1577 in the APC gene and a point mutation (V600E) in BRAF. APC is a tumor-suppressor gene and its deletion and point mutations correlate to the development of numerous types of cancers, especially colorectal cancers. BRAF with a V600E mutation is a well-established oncogene in melanoma and colorectal cancer, but it is rarely found in breast cancer. We investigated what signaling drivers were responsible for the proliferation and viability of this cell line.

### 3.2. Probing Oncogenic Protein Kinase Drivers in DU-4475 Cell Line Viability

To determine what signaling pathways are essential for the viability of DU-4475 cells, we screened it against a panel of 20 protein kinase inhibitors (PKI) (Table 2). These inhibitors included 10 PKIs against various receptor protein tyrosine kinases that have been associated with some cancer types. Other intended targets included protein kinases in the MAPK pathway, PI3K pathway, and Src kinases. Many of these kinases are suggested to be involved in TNBC development.

**Figure 1:**
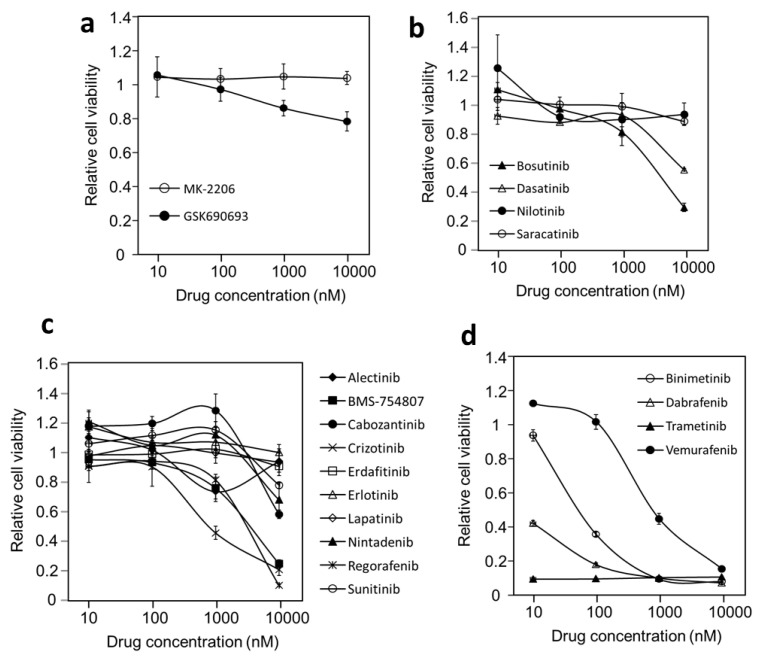
Inhibition of DU-4475 cell viability by different protein kinase inhibitors. Cell treatment and viability assays were conducted as described in the Materials and Methods. The cells were treated with each drug at four concentrations and the relative cell viability for each treatment was normalized to the control treatment of 1% DMSO. (**a**) Akt inhibitors. (**b**) Cytosolic PTK inhibitors. (**c**) Receptor PTK inhibitors. (**d**) BRAF and Mek inhibitors.

Most of the inhibitors are approved as treatments for appropriate cancers. Figure 1 shows the responses of DU-4475 to these drugs at four concentrations from 10 nM to 10 μM. DU-4475 cells did not strongly respond to Akt inhibitors (Figure 1a), Src or Abl inhibitors (Figure 1b), nor ten inhibitors against various receptor PTKs (Figure 1c). However, they were potently inhibited by a BRAF inhibitor, dabrafenib, and two Mek inhibitors, trametinib and binimetinib (Figure 1d). The most potent inhibitor was trametinib, which appeared to fully inhibit DU-4475 viability at 10 nM. These results are consistent with the GDSC1 data.

**Figure 2:**
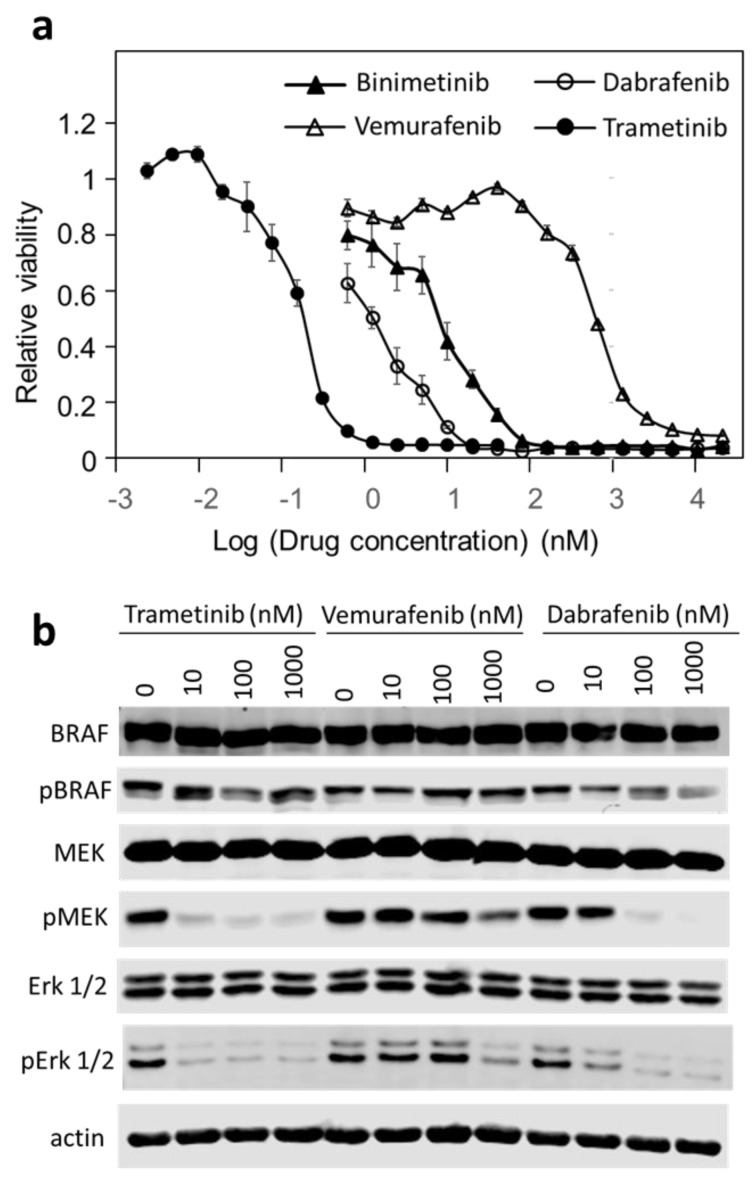
Effects of BRAF and Mek kinase inhibitors on the viability and the MAPK pathway signaling. (**a**) Dose-dependent inhibition of DU-4475 cell viability by BRAF (dabrafenib and vemurafenib) and Mek (binimetinib and trametinib) kinase inhibitors. The reported values are the average of six wells from two triplicate assays and standard error. (**b**) Western blots of BRAF, Mek and Erk total protein and phosphorylation status in DU-4475 cells after the cells were treated with indicated drugs for 1 h. β-actin was used as a loading control. Uncropped Western Blots and densitometry can be found at supplementary.

To better characterize the response of DU-4475 to these drugs, cell responses to the BRAF and Mek inhibitors at 16 concentrations were determined (Figure 2a). The dose–response data to these four drugs were fitted to the Hill equation and the inhibitory parameters are shown in Table 3. The data confirmed that DU-4475 cells are most sensitive to trametinib, followed by dabrafenib and binimetinib. The IC_50_ of 0.28 nM for trametinib is more potent in DU-4475 than previously reported inhibition of any other cancer cell line [62]. It is also notable that vemurafenib showed only mild inhibition, with an IC_50_ of 507 nM, while dabrafenib was much more potent, with an IC_50_ of 2.4 nM. All four drugs inhibited the cell viability to 95% or more (I_max_) and the inhibition displayed mild positive cooperativity in inhibiting cell viability, with the *n* values above 1. These characteristics indicate that each drug can shut down DU-4475 viability by binding to a single high-affinity target, BRAF or Mek. This result suggests that DU-4475 cells are solely dependent on BRAF signaling for viability. Considering that DU-4475 contains an oncogenic BRAF V600E mutation, these data suggest that DU-4475 is a mono-driver cancer cell line.

### 3.3. Trametinib and Dabrafenib Fully Block Mek and Erk Activation in DU-4475 

To confirm that these drugs are blocking the intended targets, we determined the effects of drug treatments on the phosphorylation states of key protein kinases in the MAP kinase pathway (Figure 2b). BRAF was phosphorylated on Ser445 in untreated cells and the phosphorylation level was not affected by any of the inhibitors. Trametinib and dabrafenib nearly fully blocked the phosphorylation of Mek (Ser217 and Ser221) and Erk (Thr202/Tyr204) at 10 nM and 100 nM, respectively, while vemurafenib only inhibited their phosphorylation at 1000 nM. These observations are consistent with the potency of each drug, inhibiting cell viability. 

It is notable that trametinib not only inhibited Erk phosphorylation by Mek, but also inhibited Mek phosphorylation by BRAF. This pattern of inhibition by trametinib is consistent with its reported mechanism of action [63,64,65]. Even though trametinib is generally referred to as an Mek inhibitor, it more potently inhibits Mek phosphorylation by BRAF than it inhibits Mek phosphorylation of Erk. This is achieved by interfering with BRAF’s ability to recognize Mek, even though it is not a general BRAF inhibitor in phosphorylating other BRAF substrates [63]. Thus, trametinib should be more accurately referred to as an inhibitor of Mek activation by BRAF rather than an inhibitor of Mek activity.

### 3.4. Blocking BRAF or Mek Inhibits Proliferation and Causes Cell Death in DU-4475

**Figure 3:**
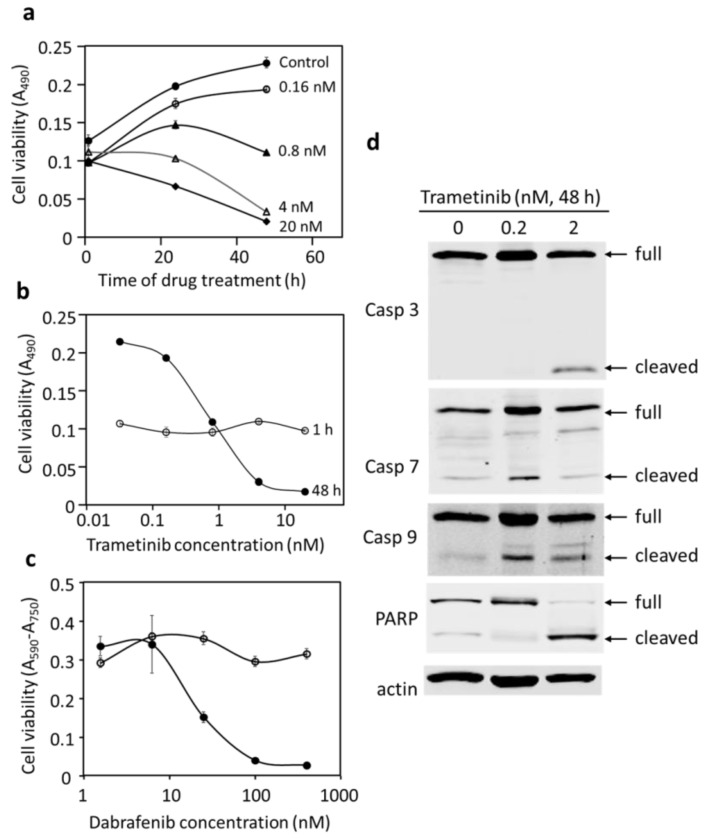
Effects of trametinib and dabrafenib on DU-4475 cell viability and death. (**a**) Growth curves of DU-4475 treated with different concentrations of trametinib over time. (**b**) Dose–response curves of DU-4475 treated with trametinib for 1 h and 48 h. (**c**) Dose–response curves of DU-4475 treated with dabrafenib for 1 h and 48 h. (**d**) Western blots of full length and cleaved caspases, PARP, and β-actin. Uncropped Western Blots and densitometry can be found at supplementary.

A cancer drug may inhibit cell proliferation and/or cause cell death. To determine the mechanism of inhibition of trametinib and dabrafenib on DU-4475, cell viability over time of treatments was monitored. Figure 3a shows the cell viability of DU-4475 at different trametinib treatments over time. The treatment with trametinib up to 20 nM for 1 h did not result in any significant changes in DU-4475 viability compared to the control. DU-4475 cells treated with 0.16 nM trametinib increased viability at 24 and 48 h. Treatments with higher concentrations of trametinib for 24 h and 48 h resulted a concentration-dependent inhibition of cell viability. Especially notable were the treatments at 4 and 20 nM, which resulted in a significant decrease in cell viability in 48 h. These results suggested that treatments with 4 and 20 nM trametinib for 48 h killed the cells. 

The data can also be viewed as DU-4475 dose–response curves for the 1 h and 48 h treatments in Figure 3b. The presence of trametinib up to 0.8 nM allowed some increase in DU-4475 viability in 48 h, indicating continued proliferation, albeit at reduced rates compared to the control. Treatment with trametinib at 4 nM and 20 nM for 48 h resulted in cell viability levels significantly below the original 1 h cell viability, indicating that the treatments induced death. The crossover of the 48 h dose–response curve from above to below the 1 h curve reveals the threshold of the drug concentration between growth inhibition to cell killing.

Blocking BRAF function with dabrafenib resulted in similar cell responses by DU-4475 (Figure 3c), killing cells in a concentration-dependent manner. The trametinib and dabrafenib results together indicate that partial blocking of BRAF or Mek resulted in an inhibition of DU-4475 proliferation, while complete inhibition of BRAF or Mek signaling fully blocked DU-4475 proliferation and caused cell killing. These results strongly suggest that DU-4475 is a mono-driver cancer cell line predominantly supported by a single oncogenic signaling driver, BRAF V600E.

We determined if the DU-4475 killing by trametinib was via apoptosis. DU-4475 cells treated with trametinib for 48 h were collected and the cleavage of apoptotic caspases and poly (ADP-ribose) polymerase (PARP) was analyzed. The cleavage of caspases activates them and initiates apoptosis. The cleavage of PARP inactivates it and suppresses DNA repair, which is also associated with apoptosis. Caspases 3, 7, and 9 were cleaved to expected sizes in response to trametinib treatments (Figure 3d). PARP was cleaved into an 89 kD fragment, which is also an indicator of apoptosis. These results suggest that blockage of the predominant driver pathway by trametinib is sufficient to inhibit cell proliferation and induce apoptosis in DU-4475 cells.

**Figure 4:**
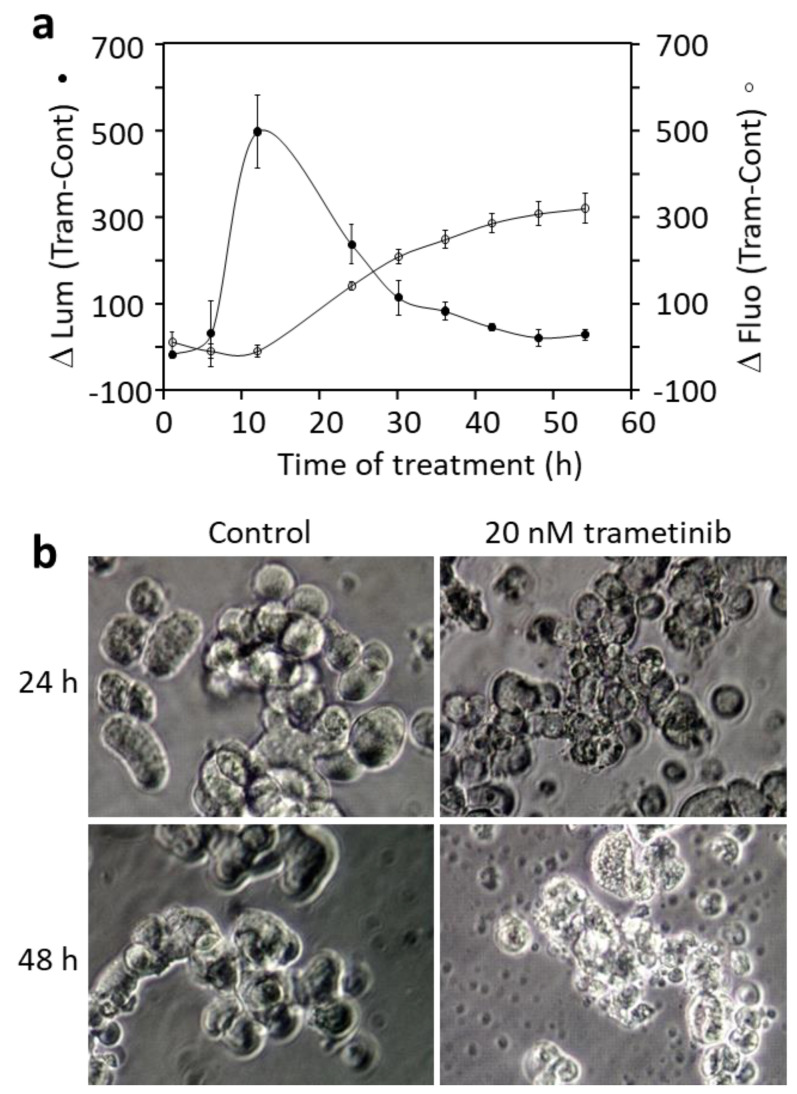
Effects of trametinib on DU-4475 cells. (**a**) Detection of apoptosis and necrosis in DU-4475 cells treated with 20 nM trametinib. DU-4475 cells were treated with 20 nM trametinib and early apoptosis and necrosis were monitored using the RealTime-Glo Annexin V Apoptosis and Necrosis Assay as described in Materials and Methods. ΔLum is the difference in luminescence of trametinib-treated cells—that of control cells. The increase in luminescence is an indication of phosphatidyl Serine translocating to the outer leaflet of the cell membrane. ΔFluo is the difference in fluorescence of a DNA-binding dye in trametinib-treated cells—those of control cells. The increase in fluorescence is an indication of cell membrane becoming permeable to the DNA binding dye. (**b**) Morphology of control and trametinib-treated DU-4475 cells.

We used the Annexin V apoptosis and necrosis assay to confirm that trametinib caused cell death in DU-4475 through apoptosis. The RealTime-Glo™ Annexin V Apoptosis and Necrosis Assay (Promega, Madison, WI, USA) measures the real-time translocation of phosphatidylserine on the outer leaflet of cell membranes during early apoptosis [57]. The assay also measures the loss of membrane integrity using a DNA-binding dye, which enters the cell and generates a fluorescent signal. Figure 4a displays the increase in the luminescence and fluorescence in DU-4475 cells due to trametinib treatment. The differential in luminescence signal between treated and control cells peaked at 11 h of treatment, an indication of early apoptosis. The subsequent loss of the luminescence signal is an indication of the loss of membrane integrity. This indicated that early apoptosis started at ~10 h treatment. The fluorescence signal increased gradually following early apoptosis, corresponding to the loss of membrane integrity due to necrosis. These results are consistent with the Western blotting results, indicating that trametinib caused apoptosis and secondary necrosis.

The morphology of the DU-4475 cells upon trametinib treatment is consistent with this result (Figure 4b). The cells in the control at 24 h or 48 h grew in suspension, displayed some loose association, and maintained a clean medium. The 20 nM trametinib-treated DU-4475 cells at 24 h displayed various degrees of apoptotic blebbing and necrosis. At 48 h, virtually all cells progressed to necrosis, with cell fragments and debris remaining. These morphological changes confirmed the Western blotting results showing the activation of caspases and degradation of PARP and the Annexin V assay results, showing apoptosis and necrosis.

### 3.5. MDA-MB-231 Is a Multi-Driver TNBC Cell Line Dependent on Both the MAP Kinase Pathway and Src Kinase for Proliferation

We next compared the inhibitory properties of DU-4475 and MDA-MB-231 based on the following two considerations. First, the remarkable potency of trametinib in blocking proliferation and inducing apoptosis in DU-4475 prompted us to examine if this drug can be used to block the proliferation of other TNBC cells with activated MAP kinase signaling. Among the available TNBC cell lines, DU-4475 is the only one with a BRAF V600E mutation; however, MDA-MB-231 contains an oncogenic KRAS G13D mutation. It has been reported that MDA-MB-231 is vulnerable to Mek inhibition by selumetinib [66,67]. Second, we previously determined that MDA-MB-231 is driven by both Src and the MAPK pathway [53], making MDA-MB-231 a multi-driver cancer cell line. A comparison between DU-4475 and MDA-MB-231 might offer insights into the multi-driver versus mono-driver oncogenic mechanisms. 

**Figure 5:**
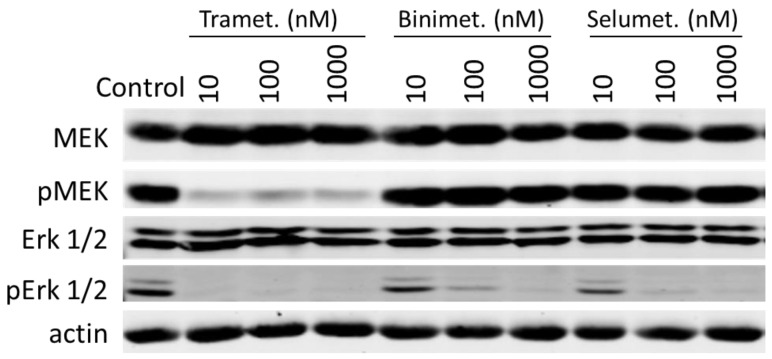
Effects of Mek inhibitors on Mek and Erk phosphorylation in MDA-MB-231 cells. MDA-MB-231 cells were treated with the indicated drugs for 1 h and the protein expression level and phosphorylation level of Mek and Erk were determined by Western blotting. Uncropped Western Blots and densitometry can be found at supplementary.

We compared the inhibition of MDA-MB-231 by different Mek inhibitors and dasatinib, an Src inhibitor (Table 4). Consistent with our previous report on MDA-MB-231, individual Mek inhibitors, trametinib, selumetinib, or binimetinib, are not potent inhibitors of MDA-MB-231, reaching only a maximal inhibition of 43% for selumetinib and somewhat less for the other two inhibitors. However, even with partial inhibition, trametinib displayed a better potency (K_d_ = 13 nM) than the other Mek inhibitors (K_d_ of 563 nM and 199 nM for selumetinib and binimetinib, respectively) at achieving the maximal inhibition. The lack of full inhibition of MDA-MB-231 cell viability by these inhibitors is not due to restricted cell permeability nor failure to bind to their intended targets. Each drug effectively inhibited the phosphorylation and activation of Erk, trametinib at 10 nM and both binimetinib and selumetinib at 100 nM (Figure 5). Consistent with trametinib being an inhibitor of Mek activation by BRAF, trametinib also inhibited Mek phosphorylation, while binimetinib and selumetinib did not. These results suggest that blocking Mek and Erk in the MAP kinase pathway is not sufficient to block the viability of MDA-MB-231 cells. This suggestion is consistent with MDA-MB-231 being a multi-driver cancer cell line.

**Figure 6:**
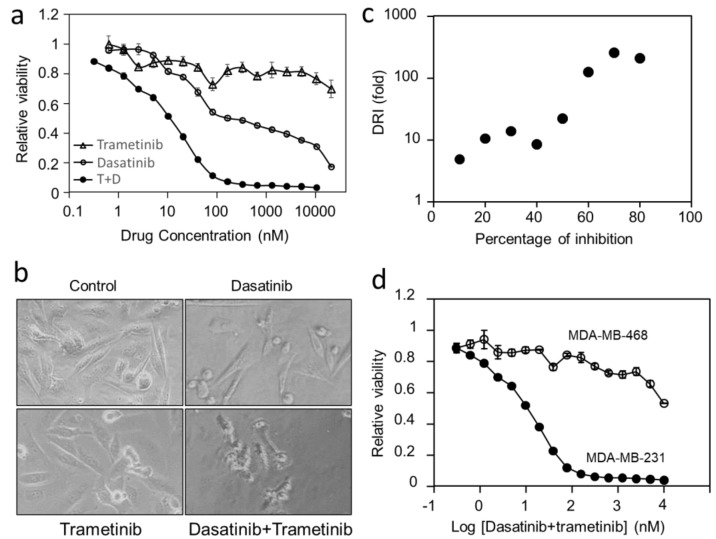
Effects of dasatinib, trametinib, and their combination on the viability and morphology of MDA-MB-231 cells. (**a**) Cell viability was determined after MDA-MB-231 cells were treated with dasatinib, trametinib, and their combination at equimolar concentrations for 48 h. (**b**) The relationship between the dose-reduction index (DRI) and the percentage of inhibition. (**c**) MDA-MB-231 cell morphology at the end of treatment with dasatinib, trametinib, and their combination (1 μM). (**d**) Comparison between MDA-MB-231 and MDA-MB-468 in the dose–response to dasatinib/trametinib combination.

Dasatinib inhibited MDA-MB-231 in a biphasic manner, with the first phase accounting for 55% of cell viability and a K_d1_ of 27 nM. This pattern is similar to what has been previously reported [53]. These analyses confirmed that the MAP kinase pathway and Src are independent drivers in MDA-MB-231. Thus, we tested the effects of dasatinib and trametinib combination on MDA-MB-231 viability. The combination of equal concentrations was an extremely potent inhibitor cocktail for this cell line, with an IC_50_ of 8.2 nM (Figure 6a and Table 4), about 10-fold more potent than dasatinib + selumetinib or dasatinib + binimetinib combinations (Table 4). At the end of 48 h treatments, dasatinib (100 nM) and trametinib (100 nM)-treated cells displayed similar morphology to control cells. In contrast, the combination-treated cells were killed, with some cell debris remaining (Figure 6b).

Especially remarkable is the incredible synergy between dasatinib and trametinib at inhibiting MDA-MB-231 cell viability. The combination reached near-complete inhibition of MDA-MB-231 viability at 100 nM, while either drug alone did not reach that level of inhibition at 20 μM. The synergy is strong at all inhibition levels and it becomes especially striking at 60% or higher levels of inhibition. For example, the IC_70_ (drug concentration for 70% inhibition) is 25 nM for the combination, 12.6 μM for dasatinib and above 20 μM for trametinib. These numbers translate into a combination index (CI) below 0.0039 and a dose-reduction index (DRI) above 257 [55,68] (Figure 6c). Thus, the drug combination is >250-fold more potent than dasatinib and trametinib combined if there was no synergy. This combination offers the most potent targeted drug combination for this cell line. The potency is even more favorable than some targeted drugs for mono-driver cancers approved for clinical applications.

To determine if the dasatinib/trametinib combination is specific for MDA-MB-231 cells, we compared the inhibition of MDA-MB-231 to that of MDA-MB-468 (Figure 6d). MDA-MB-468 has been shown to overexpress EGFR and lack PTEN and it is sensitive to inhibition by the combination of lapatinib and GSK-690693. MDA-MB-468 cells are >1000-times more resistant to the combination of dasatinib and trametinib, with an IC_50_ above 10 μM. This result demonstrates that the sensitivity of MDA-MB-231 to the dasatinib/trametinib combination is determined by the unique oncogenic driving mechanism in this cell.

### 3.6. Blocking Each Driver Partially Inhibits Cell Proliferation, While Blocking Both Drivers Induces Apoptosis in MDA-MB-231

**Figure 7:**
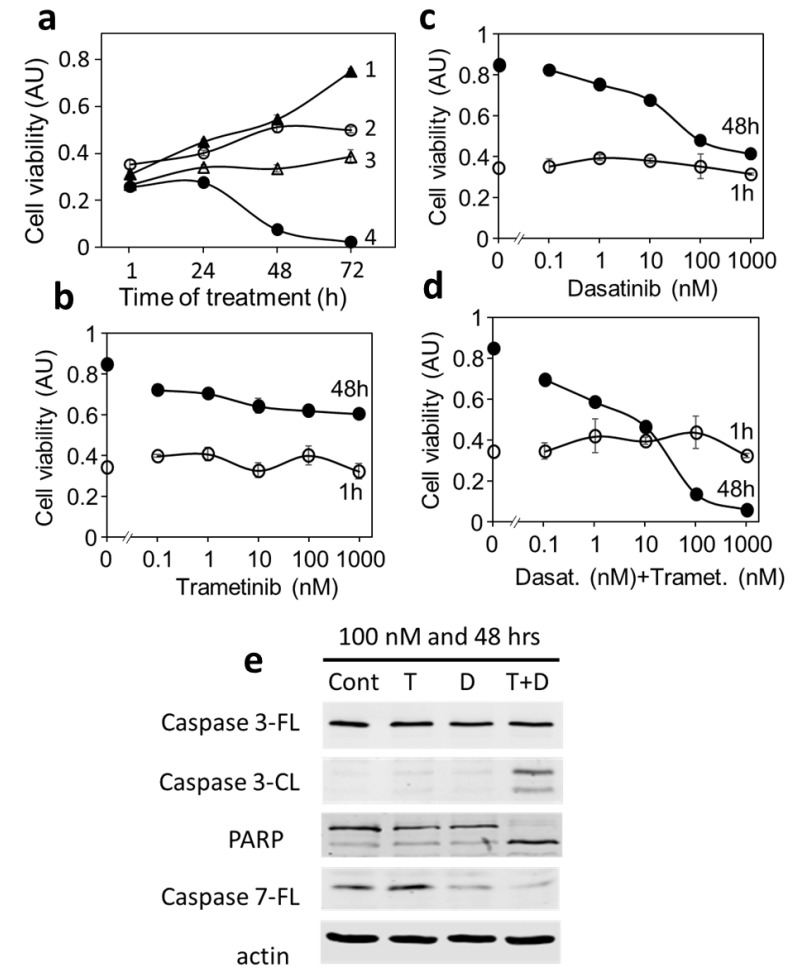
Time- and concentration-dependent effects of dasatinib, trametinib, and their combination on the growth and survival of MDA-MB-231 cells. (**a**) Growth curves of MDA-MB-231 control cells (1) under the treatment of 1 μM trametinib (2), dasatinib (3), or the trametinib + dasatinib combination (4). (**b**) Dose-dependent effects of 1 h and 48 h treatments with trametinib on MDA-MB-231 cells. (**c**) Dose-dependent effects of 1 h and 48 h treatments with dasatinib on MDA-MB-231 cells. (**d**) Dose-dependent effects of 1 h and 48 h treatments with trametinib + dasatinib on MDA-MB-231 cells. (**e**) Effects of 100 nM trametinib (T), dasatinib (D), and their combination (T + D) on the cleavage of caspases 3 and 7 and PARP, determined by Western blots. Uncropped Western Blots and densitometry can be found at supplementary.

The strong synergy between dasatinib and trametinib prompted us to further investigate the mechanism of the drug combination. Figure 7a displays the growth curves of MDA-MB-231 in the presence of 1 μM trametinib, dasatinib, and their combination. Dasatinib or trametinib alone at 1 μM resulted in a slower increase in cell viability up to 72 h, but neither decreased cell viability. The combination of dasatinib and trametinib at 1 μM resulted in a slight increase in cell viability up to 24 h and a dramatic decrease in cell viability in 48 h and 72 h, indicating that the combination resulted in near-complete cell killing (Figure 7a).

We next determined the concentration-dependent effects of trametinib, dasatinib, and the combination on cell viability in 48 h treatments (Figure 7b–d). Treatment with trametinib for 48 h resulted in a dose–response curve above the 1 h treatment curve at all concentrations (Figure 7b), indicating that trametinib only mildly inhibited cell growth but did not kill the cells up to 1 μM. Similarly, the 48 h dose–response curves for dasatinib were above the 1 h treatment at all concentrations (Figure 7c), indicating that dasatinib inhibited cell growth but did not kill the cells up to 1 μM. In contrast, the dose–response curve for the 48 h combination treatment was above the 1 h curve below 10 nM and crossed over to below the 1 h curve at 100 and 1000 nM (Figure 7d). This cross-over pattern indicated that the number of viable cells after 48 h treatment with the drug combination dropped below that of the amount observed at 1 h, indicating that cells were killed in the combination but not with trametinib or dasatinib alone. Western blotting (Figure 7e) demonstrated that the drug combination at 100 nM caused significant cleavage of caspases 3, 7, and PARP, indicating cell death is through apoptosis.

The increase in apoptosis when both pathways are blocked also provides a mechanistic explanation for the dramatic synergy observed earlier. When only one driver is blocked by a drug, the cells are still able to survive or even grow with the support of the other pathway. When both drivers are blocked, the cells lose all growth stimulation and initiate apoptosis. This is in direct contrast with that of DU-4475, for which apoptosis is induced by one drug. These results suggest that a drug blocking one driver can kill mono-driver cancer cells, but a multi-driver cancer cell can be killed only by a drug combination blocking both signaling pathways.

## 4. Discussion

Despite dramatic progress in targeted cancer therapies blocking oncogenic signaling, only a relatively small number of cancers have benefitted from effective therapy and most, if not all, effectively treated cancers are mono-driver cancers. Most cancers are dependent on multiple drivers, making them naturally resistant to treatments targeting any single driver. Triple-negative breast cancers appear to be mostly multi-driver cancers. Understanding the molecular basis of multi-driver versus mono-driver oncogenesis is a major challenge for developing targeted therapies for TNBC.

### 4.1. DU-4475 as a Mono-Driver Cancer Cell Model

This study provides evidence that DU-4475 is a mono-driver cancer cell line. DU-4475 cells harbor a BRAF V600E mutation, but the functional significance of this mutation in this cell line is not fully clear. While BRAF V600E is a predominant driver in some cells, especially in melanoma, other cells with this mutation are not entirely dependent on it for oncogenic proliferation. In DU-4475, blocking BRAF or Mek kinase alone is sufficient to completely block cell viability and cause apoptosis (Figure 2, Figure 3 and Figure 4). These results suggest that BRAF V600E-activated MAP kinase signaling is essential for the survival of DU-4475 cells and blocking this pathway at either step is sufficient to kill DU-4475 cells. To our knowledge, this is the only TNBC cell line that is dependent on a single oncogenic driver, i.e., a mono-driver cancer cell line.

The inhibition of a mono-driver cancer cell line by the inhibitor for its driver displays three characteristics when the dose–response data are fitted into the Hill equation: an IC_50_ consistent with the target kinase inhibition, an I_max_ close to 100%, and a Hill slope, *n*, around 1. When a cell response to a given PKI meets these criteria, the target kinase of the drug can be considered the predominant driver and the cancer cell can be considered a mono-driver cancer cell. DU-4475 cells meet these criteria as they are potently and fully inhibited by BRAF or Mek inhibitors, demonstrating the cells’ dependence on BRAF V600E for cell viability. The most noteworthy drug is trametinib, which inhibited DU-4475 cells with an IC_50_ of 0.28 nM and reached 95% inhibition at about 1 nM. It can be expected that cancer with a similar mechanism would be susceptible to targeted therapy with trametinib.

### 4.2. BRAF V600E as a Therapeutic Target in TNBC

A BRAF mutation is rare in breast cancer and TNBC, but several-BRAF V600E-driven TNBC cases have been reported. Pircher et al. reported [69] that a 38-year-old female TNBC patient with a BRAF V600E mutation developed multiple lung metastases. The patient was treated with vemurafenib, which resulted in clear radiological improvements within three months. At the time of the report (19 months after the therapy started), the lung metastases remain radiologically stable and the patient remains in good clinical condition. The second case of a TNBC patient with a BRAF V600E mutation was reported by Wang et al. [70]. This 60-year-old patient was first treated with chemotherapy to achieve a 7-month progression-free survival (PFS). The patient received vemurafenib and albumin-bound paclitaxel as a second-line therapy, exhibiting regression of some metastatic pulmonary lesions with concomitant progression of other lesions and achieved a 4.4-month PFS. Genetic testing of the progressed pulmonary lesion revealed the presence of the BRAF V600E mutation together with other newly acquired mutations in PDGFRB, NF2, GRM3, MLH1, FOXA1, LRP1B, and amplification of androgen receptor. The patient ultimately died of multiple organ failure and achieved 12 months of overall survival. The third TNBC patient with a BRAF V600E mutation was a 57-year-old woman with metastatic TNBC and chemotherapy-refractory massive pleural effusion [71]. She also had oncogenic PIK3CA H1047R mutation, among others. After failure with anthracycline- and taxane-based chemotherapy and palliative radiotherapy, the patient was treated with a dabrafenib and trametinib combination. The patient exhibited improved conditions with decreases in swelling and pain, a decrease in pleural fusion, and a reduction in the size of the axillary lymph nodes for several weeks. However, the patient developed another subcutaneous tumor and died 12 weeks after initiating the dabrafenib/trametinib treatment. All three case reports support BRAF V600E as a valid treatment target in TNBC. These case studies also make it clear that the acquisition of additional oncogenic driver mutations would make it ineffective to treat the patient with a BRAF inhibitor alone and finding effective drug combinations that block all oncogenic drivers would be necessary. The presence of the H1047R mutation in PIK3CA, a well-established oncogenic driver [72], in the third patient, suggests that the PI 3-kinase pathway is also activated in this patient, likely making it a multi-driver cancer. A drug combination blocking both BARF and the PI 3-kinase pathway, such as PI 3-kinase inhibitors or Akt inhibitors, may have been more effective than the dabrafenib and trametinib combination.

### 4.3. Blocking Both Oncogenic Drivers in MDA-MB-231 Causes Synthetic Lethality

Most cancers are likely dependent on multiple oncogenic drivers. We previously demonstrated that several colorectal cancers and TNBC cell lines are multi-driver cancer cells, including MDA-MB-231. The identification of a mono-driver cancer cell model in DU-4475 enabled us to compare the drug response patterns of a mono-driver versus a multi-driver cancer cell model. While blocking the predominant driver in a mono-driver cancer cell line is sufficient to fully inhibit cell proliferation and cause cell death, blocking a driver in a multi-driver cancer model only causes partial inhibition to cancer viability and does not kill the cancer cells. The MDA-MB-231 cells are dependent on the MAP kinase pathway and Src signaling for survival and blocking either the MAP kinase pathway or Src only partially inhibits cell viability and without causing cell death. Only the combination of Src and Mek inhibitors completely inhibits cell proliferation and causes programmed cell death. This crucial difference between mono-driver and multi-driver cancer cells provides an explanation why single-agent targeted therapy only works well for mono-driver cancer cells but not for multi-driver cancer cells and supports combination targeted therapy as a viable strategy against multi-driver cancers.

The drug combination is also strikingly synergistic. Dasatinib displays a biphasic inhibition with a potent phase 1 (F_1_ = 55% and K_d1_ = 27 nM) and an F_2_ of 45% with K_d2_ of 18 μM. With both phases combined, dasatinib is still not an effective inhibitor, reaching 70% inhibition at 12.5 μM. Trametinib alone is even less effective, only causing a maximal inhibition of ~20%. However, the combination is much more potent, with an IC_50_ of 8.2 nM, and achieved near-complete inhibition at about 100 nM. The strong synergy is also reflected in the CI of <0.003 and DRI > 300. This striking synergy can be explained by the multi-driver oncogenic mechanism. Because the cells are supported by two pathways, blocking either Src or the MAP kinase pathway alone still leaves the cells viable and only blocking both pathways causes cell death. The striking synergy supports the potential effectiveness of combination targeted therapy for multi-driver cancers.

### 4.4. Single-Drug Lethality versus Synthetic Lethality

An ideal cancer drug should inhibit cancer cell growth and cause cancer cell lethality with specificity. Our data demonstrate that for mono-driver cancer cells, such as DU-4475, the specific lethality is achieved with a single drug at low nM concentrations. This is presumably the underlying basis for the success of targeted therapies against mono-driver cancers. Because multi-driver cancer cells can survive with the support of multiple drivers, pharmacological lethality is not achievable with any one targeted drug blocking any driver and is only achievable when a drug combination blocking all drivers is used. Combinational inhibition of MDA-MD-231 can be considered a form of pharmacological synthetic lethality. This pharmacological synthetic lethality is rooted in the multi-driver nature of MDA-MB-231 cells, as they have acquired an overlapping dependence on the MAP kinase pathway and Src kinases. The partial functional redundancy between these two pathways likely makes MDA-MB-231 cells much more resilient and gives them a significant growth advantage and adaptability. The advantage of this multi-driver overlapping mechanism to the cancer cells is evident and likely applicable to other multi-driver cancer cells. Further research is needed to establish this as a broadly utilized mechanism.

Traditionally, synthetic lethality is defined at the genetic level, where the loss-of-function mutations in two genes cause lethality [73,74], while the loss of either gene alone is not lethal. This concept was further expanded to include the lethality caused by genetic mutation in one gene and chemical inhibition of another. For example, BRCA1 and BRCA2 mutations and PARP inhibitors are synthetically lethal, which has become the conceptual basis for the new therapeutic paradigm of PARP inhibition for cancer patients carrying BRCA1 and BRCA2 mutations [75]. The pharmacological synthetic lethality between dasatinib and trametinib observed in MDA-MB-231 cells carries this concept a step further. This concept could provide a molecular basis for combination targeted therapies in multi-driver cancers.

## 5. Conclusions

Triple-negative breast cancer is notoriously heterogeneous, with different patients using different genetic and biochemical mechanisms of oncogenesis. This heterogeneity is clearly illustrated in the two cancer cell lines used in this study. Even though both DU-4475 and MDA-MB-231 have mutations in the MAP kinase pathway, BRAF V600E in DU-4475 and KRAS G13D in MDA-MB-231, these two cell lines have one critical difference. While DU-4475 is a mono-driver cancer cell line, solely dependent on BRAF for survival and proliferation, MDA-MB-231 relies on both the MAP kinase pathway and Src signaling. Comparing these cell lines offers critical insights into mono-driver versus multi-driver oncogenesis and strategies for blocking them. 

The results demonstrated that DU-4475 cells, as mono-driver cancer cells, can be blocked and killed by BRAF or Mek inhibitors. The MDA-MB-231 cells, in contrast, can still survive when either the MAP kinase pathway or Src kinases are fully inhibited. The cells can only be killed when both pathways are blocked. This pharmacological synthetic lethality offers an underlying basis for killing multi-driver cancer cells by targeted drug combinations. In both cases, cell killing was achieved through apoptosis. These results explain why successful targeted therapies are against mono-driver cancers and offer a proof of concept for a mechanism-based combination targeted therapy strategy for multi-driver cancers. It is well observed that broadly targeting protein kinase inhibitors are generally more toxic toward more cells than those with narrow inhibitory specificity. However, such broadly targeted drugs tend to cause general toxicity. Identifying the oncogenic drivers and drug combinations to specifically block the oncogenic drivers through combination therapy is a plausible precision approach to achieve multi-target inhibition without causing general toxicity. Further validation is needed to determine if this strategy is broadly applicable. While most TNBC cancers are expected to be multi-driver cancers, the functional signaling drivers have yet to be identified in most TNBC models. The elucidation of signaling drivers in other TNBC cell models will further validate this strategy.

## Figures and Tables

**Table 1:** TNBC cell lines and their most potent signaling protein kinase inhibitors in the Genomics of Drug Sensitivity in Cancer (GDSC1) database.

Cell Line	Most Potent PKI	Target	IC_50_ (nM)	Z-Score
BT-20	AKT inhibitor III	Akt	3505	−1
BT-549	GW441756	NTRK1	3036	−1.3
DU-4475	Dabrafenib	BRAF	6.3	−3.5
DU-4475	Trametinib	Mek	0.5	−2.1
HCC1143	Jak3_7406	Jak3	6312	−1.3
HCC1187	GW441756	NTRK1	89.7	−4.7
HCC1395	Panopanib	CSF1R, KIT, PDGFR	2432	−1.7
HCC1599	Jak1_3715	Jak 1	2185	−3.9
HCC1806	GSK1904529A	IGF1R, IR	3466	−1.4
HCC1937	WZ3105	Src, Rock2, NTRK2, FLT3	273	−0.8
HCC38	AKT inhibitor III	Akt 1, 2, 3	2085	−1.6
HCC70	AKT inhibitor III	Akt 1, 2, 3	775	−2.6
HCC70	MK-2206	Akt 1, 2	1245	−2.2
Hs-578-T	AT7868	Akt	1160	−2
MDA-MB-157	Motesanib	VEGFR, RET, KIT, PDGFR	6243	−0.8
MDA-MB-231	Alectinib	Alk	4639	−2
MDA-MB-436	GW441756	NTRK1	5057	−0.8
MDA-MB-453	FGFR_0939	FGFR4	643	−2.6
MDA-MB-468	Amuvatinib	Kit, PDGFRA, FLT3	1267	−2.2

**Table 2:** Protein kinase inhibitors used to probe the oncogenic signaling mechanism of DU-4475.

Inhibitor	Main Target Kinase	Signaling Pathway
Alectinib	Alk, Ret	Receptor PTKs
BMS-754807	Insulin receptor, IGF-1R	Receptor PTKs
Cabozantinib	Tet, VEGFR	Receptor PTKs
Crizotinib	Alk, Ros1, Met	Receptor PTKs
Erdafitinib	FGFR	Receptor PTKs
Erlotinib	EGFR	Receptor PTKs
Lapatinib	EGFR	Receptor PTKs
Nintadenib	VEGFR, PDGFR, FGFR	Receptor PTKs
Regorafenib	VEGFR	Receptor PTKs
Sunitinib	PDGFR, VEGFR	Receptor PTKs
MK-2206	Akt	PI 3-Kinase pathway
GSK690693	Akt	PI 3-kinase pathway
Vemurafenib	BRAF	MAP kinase pathway
Dabrafenib	BRAF	MAP kinase pathway
Trametinib	Mek	MAP kinase pathway
Binimetinib	Mek	MAP kinase pathway
Bosutinib	Src, Abl	Cytoplasmic PTKs
Dasatinib	Src, Abl	Cytoplasmic PTKs
Saracatinib	Src, Abl	Cytoplasmic PTKs
Nilotinib	Abl	Cytoplasmic PTKs

**Table 3:** Inhibition ^1^ parameters of BRAF and Mek inhibitors on DU-4475 cells.

Inhibitor	Target Kinase	IC_50_ (nM)	I_max_ (%)	*n*
Dabrafenib	BRAF	2.4 ± 0.5	98.0 ± 0.25	1.35 ± 0.2
Vemurafenib	BRAF	507 ± 16	95.0 ± 0.9	1.29 ± 0.07
Trametinib	Mek	0.28 ± 0.03	96.2 ± 0.35	1.76 ± 0.23
Binimetinib	Mek	7.3 ± 1.5	96.4 ± 0.8	1.50 ± 0.34

^1^ The inhibitory parameters were calculated by fitting 16 concentration dose–response data to the Hill equation as described in Materials and Methods. Average and standard errors were calculated from six sets of data from two triplicate assays.

**Table 4:** Inhibition ^1^ of MDA-MB-231 by individual and combination PKIs.

Inhibitor	Hill Analysis			Biphasic Analysis			
	IC_50_ (nM)	*n*	I_max_ (%)	F_1_ (%)	K_d1_ (nM)	F_2_ (%)	K_d2_ (μM)
Dasatinib	66 ± 8.5	0.65 ± 0.03	73 ± 1.3	55 ± 0.8	27 ± 3.3	45 ± 0.8	18 ± 0.5
Trametinib	13 ± 1.3	0.97 ± 0.03	26 ± 3.1	22 ± 1.9	7.7 ± 2.9	79 ± 1.9	>100
Binimetinib	199 ± 5.8	0.92 ± 0.07	19 ± 0.5	16 ± 0.5	125 ± 21	84 ± 0.5	>100
Selumetinib	563 ± 172	0.74 ± 0.11	43 ± 4.7	28 ± 3.8	156 ± 55	72 ± 3.9	97 ± 13
Dasa + Tram	8.2 ± 0.3	0.75 ± 0.03	98 ± 0.4	ND	ND	ND	ND
Dasa + Bini	64 ± 2.9	0.90 ± 0.03	99 ± 0.5	ND	ND	ND	ND
Dasa + Selu	78 ± 11	0.71 ± 0.04	94 ± 0.3	ND	ND	ND	ND

^1^ These inhibitory parameters were obtained by analyzing dose response data at 16 concentrations by the Hill equation and the biphasic analysis as described in Materials and Methods.

## Data Availability

Data supporting the reported results are provided in the Supplemental Data or available upon request.

## Supplementary Materials

The following supporting information can be downloaded at: https://www.mdpi.com/article/10.3390/cancers14164027/s1, Uncropped Western Blots and densitometry scanning data.

## References

[1] DeSantis C.E., Ma J., Gaudet M.M., Newman L.A., Miller K.D., Goding Sauer A., Jemal A., Siegel R.L. (2019). Breast cancer statistics, 2019. CA Cancer J. Clin..

[2] Siegel R.L., Miller K.D., Jemal A. (2020). Cancer statistics, 2020. CA Cancer J. Clin..

[3] Network C.G.A. (2012). Comprehensive molecular portraits of human breast tumours. Nature.

[4] Ciriello G., Gatza M.L., Beck A.H., Wilkerson M.D., Rhie S.K., Pastore A., Zhang H., McLellan M., Yau C., Kandoth C. (2015). Comprehensive Molecular Portraits of Invasive Lobular Breast Cancer. Cell.

[5] Foulkes W.D., Smith I.E., Reis-Filho J.S. (2010). Triple-negative breast cancer. N. Engl. J. Med..

[6] Bianchini G., Balko J.M., Mayer I.A., Sanders M.E., Gianni L. (2016). Triple-negative breast cancer: Challenges and opportunities of a heterogeneous disease. Nat. Rev. Clin. Oncol..

[7] Boyle P. (2012). Triple-negative breast cancer: Epidemiological considerations and recommendations. Ann. Oncol..

[8] Gupta G.K., Collier A.L., Lee D., Hoefer R.A., Zheleva V., van Reesema L.L.S., Tang-Tan A.M., Guye M.L., Chang D.Z., Winston J.S. (2020). Perspectives on Triple-Negative Breast Cancer: Current Treatment Strategies, Unmet Needs, and Potential Targets for Future Therapies. Cancers.

[9] Pal S.K., Childs B.H., Pegram M. (2011). Triple negative breast cancer: Unmet medical needs. Breast Cancer Res. Treat..

[10] Schroeder M.C., Rastogi P., Geyer C.E., Miller L.D., Thomas A. (2018). Early and Locally Advanced Metaplastic Breast Cancer: Presentation and Survival by Receptor Status in Surveillance, Epidemiology, and End Results (SEER) 2010–2014. Oncologist.

[11] Howlader N., Cronin K.A., Kurian A.W., Andridge R. (2018). Differences in Breast Cancer Survival by Molecular Subtypes in the United States. Cancer Epidemiol. Biomark. Prev..

[12] Thomas A., Rhoads A., Pinkerton E., Schroeder M.C., Conway K.M., Hundley W.G., McNally L.R., Oleson J., Lynch C.F., Romitti P.A. (2019). Incidence and Survival Among Young Women with Stage I-III Breast Cancer: SEER 2000-2015. JNCI Cancer Spectr..

[13] Carey L., Winer E., Viale G., Cameron D., Gianni L. (2010). Triple-negative breast cancer: Disease entity or title of convenience?. Nat. Rev. Clin. Oncol..

[14] Newman L.A., Kaljee L.M. (2017). Health Disparities and Triple-Negative Breast Cancer in African American Women: A Review. JAMA Surg..

[15] Iqbal J., Ginsburg O., Rochon P.A., Sun P., Narod S.A. (2015). Differences in breast cancer stage at diagnosis and cancer-specific survival by race and ethnicity in the United States. JAMA.

[16] Murphy C.C., Bartholomew L.K., Carpentier M.Y., Bluethmann S.M., Vernon S.W. (2012). Adherence to adjuvant hormonal therapy among breast cancer survivors in clinical practice: A systematic review. Breast Cancer Res. Treat..

[17] Puhalla S., Bhattacharya S., Davidson N.E. (2012). Hormonal therapy in breast cancer: A model disease for the personalization of cancer care. Mol. Oncol..

[18] Figueroa-Magalhães M.C., Jelovac D., Connolly R., Wolff A.C. (2014). Treatment of HER2-positive breast cancer. Breast.

[19] Waks A.G., Winer E.P. (2019). Breast Cancer Treatment: A Review. JAMA.

[20] Goetz M.P., Gradishar W.J., Anderson B.O., Abraham J., Aft R., Allison K.H., Blair S.L., Burstein H.J., Dang C., Elias A.D. (2019). NCCN Guidelines Insights: Breast Cancer, Version 3.2018. J. Natl. Compr. Cancer Netw..

[21] Andreopoulou E., Schweber S.J., Sparano J.A., McDaid H.M. (2015). Therapies for triple negative breast cancer. Expert Opin. Pharmacother..

[22] Isakoff S.J., Mayer E.L., He L., Traina T.A., Carey L.A., Krag K.J., Rugo H.S., Liu M.C., Stearns V., Come S.E. (2015). TBCRC009: A Multicenter Phase II Clinical Trial of Platinum Monotherapy with Biomarker Assessment in Metastatic Triple-Negative Breast Cancer. J. Clin. Oncol..

[23] American Cancer Society Triple-Negative Breast Cancer. https://www.cancer.org/cancer/breast-cancer/about/types-of-breast-cancer/triple-negative.html..

[24] Berger E.R., Park T., Saridakis A., Golshan M., Greenup R.A., Ahuja N. (2021). Immunotherapy Treatment for Triple Negative Breast Cancer. Pharmaceuticals.

[25] Marquart J., Chen E.Y., Prasad V. (2018). Estimation of the Percentage of US Patients with Cancer Who Benefit from Genome-Driven Oncology. JAMA Oncol..

[26] Hanahan D., Weinberg R.A. (2011). Hallmarks of cancer: The next generation. Cell.

[27] Hanahan D., Weinberg R.A. (2000). The hallmarks of cancer. Cell.

[28] Druker B.J. (2002). Perspectives on the development of a molecularly targeted agent. Cancer Cell.

[29] Baudino T.A. (2015). Targeted Cancer Therapy: The Next Generation of Cancer Treatment. Curr. Drug Discov. Technol..

[30] Jakhetiya A., Garg P.K., Prakash G., Sharma J., Pandey R., Pandey D. (2016). Targeted therapy of gastrointestinal stromal tumours. World J. Gastrointest. Surg..

[31] Lorentzen H.F. (2019). Targeted therapy for malignant melanoma. Curr. Opin. Pharmacol..

[32] Lynch T.J., Bell D.W., Sordella R., Gurubhagavatula S., Okimoto R.A., Brannigan B.W., Harris P.L., Haserlat S.M., Supko J.G., Haluska F.G. (2004). Activating mutations in the epidermal growth factor receptor underlying responsiveness of non-small-cell lung cancer to gefitinib. N. Engl. J. Med..

[33] Roskoski R. (2020). Properties of FDA-approved small molecule protein kinase inhibitors: A 2020 update. Pharmacol. Res..

[34] Baselga J., Im S.A., Iwata H., Cortés J., De Laurentiis M., Jiang Z., Arteaga C.L., Jonat W., Clemons M., Ito Y. (2017). Buparlisib plus fulvestrant versus placebo plus fulvestrant in postmenopausal, hormone receptor-positive, HER2-negative, advanced breast cancer (BELLE-2): A randomised, double-blind, placebo-controlled, phase 3 trial. Lancet Oncol..

[35] Di Leo A., Johnston S., Lee K.S., Ciruelos E., Lønning P.E., Janni W., O’Regan R., Mouret-Reynier M.A., Kalev D., Egle D. (2018). Buparlisib plus fulvestrant in postmenopausal women with hormone-receptor-positive, HER2-negative, advanced breast cancer progressing on or after mTOR inhibition (BELLE-3): A randomised, double-blind, placebo-controlled, phase 3 trial. Lancet Oncol..

[36] André F., Ciruelos E., Rubovszky G., Campone M., Loibl S., Rugo H.S., Iwata H., Conte P., Mayer I.A., Kaufman B. (2019). Alpelisib for PIK3CA-Mutated, Hormone Receptor-Positive Advanced Breast Cancer. N. Engl. J. Med..

[37] Martín M., Chan A., Dirix L., O’Shaughnessy J., Hegg R., Manikhas A., Shtivelband M., Krivorotko P., Batista López N., Campone M. (2017). A randomized adaptive phase II/III study of buparlisib, a pan-class I PI3K inhibitor, combined with paclitaxel for the treatment of HER2- advanced breast cancer (BELLE-4). Ann. Oncol..

[38] Banerji U., Dean E.J., Pérez-Fidalgo J.A., Batist G., Bedard P.L., You B., Westin S.N., Kabos P., Garrett M.D., Tall M. (2018). A Phase I Open-Label Study to Identify a Dosing Regimen of the Pan-AKT Inhibitor AZD5363 for Evaluation in Solid Tumors and in PIK3CA-Mutated Breast and Gynecologic Cancers. Clin. Cancer Res..

[39] Saura C., Roda D., Roselló S., Oliveira M., Macarulla T., Pérez-Fidalgo J.A., Morales-Barrera R., Sanchis-García J.M., Musib L., Budha N. (2017). A First-in-Human Phase I Study of the ATP-Competitive AKT Inhibitor Ipatasertib Demonstrates Robust and Safe Targeting of AKT in Patients with Solid Tumors. Cancer Discov..

[40] Kim S.B., Dent R., Im S.A., Espié M., Blau S., Tan A.R., Isakoff S.J., Oliveira M., Saura C., Wongchenko M.J. (2017). Ipatasertib plus paclitaxel versus placebo plus paclitaxel as first-line therapy for metastatic triple-negative breast cancer (LOTUS): A multicentre, randomised, double-blind, placebo-controlled, phase 2 trial. Lancet Oncol..

[41] Hashimoto K., Tsuda H., Koizumi F., Shimizu C., Yonemori K., Ando M., Kodaira M., Yunokawa M., Fujiwara Y., Tamura K. (2014). Activated PI3K/AKT and MAPK pathways are potential good prognostic markers in node-positive, triple-negative breast cancer. Ann. Oncol..

[42] Giltnane J.M., Balko J.M. (2014). Rationale for targeting the Ras/MAPK pathway in triple-negative breast cancer. Discov. Med..

[43] Bartholomeusz C., Xie X., Pitner M.K., Kondo K., Dadbin A., Lee J., Saso H., Smith P.D., Dalby K.N., Ueno N.T. (2015). MEK Inhibitor Selumetinib (AZD6244; ARRY-142886) Prevents Lung Metastasis in a Triple-Negative Breast Cancer Xenograft Model. Mol. Cancer Ther..

[44] Nagaria T.S., Shi C., Leduc C., Hoskin V., Sikdar S., Sangrar W., Greer P.A. (2017). Combined targeting of Raf and Mek synergistically inhibits tumorigenesis in triple negative breast cancer model systems. Oncotarget.

[45] Zawistowski J.S., Bevill S.M., Goulet D.R., Stuhlmiller T.J., Beltran A.S., Olivares-Quintero J.F., Singh D., Sciaky N., Parker J.S., Rashid N.U. (2017). Enhancer Remodeling during Adaptive Bypass to MEK Inhibition Is Attenuated by Pharmacologic Targeting of the P-TEFb Complex. Cancer Discov..

[46] Nakai K., Hung M.C., Yamaguchi H. (2016). A perspective on anti-EGFR therapies targeting triple-negative breast cancer. Am. J. Cancer Res..

[47] Jafarian A.H., Kooshkiforooshani M., Farzad F., Mohamadian Roshan N. (2019). The Relationship between Fibroblastic Growth Factor Receptor-1 (FGFR1) Gene Amplification in Triple Negative Breast Carcinomas and Clinicopathological Prognostic Factors. Iran J. Pathol..

[48] Perez-Garcia J., Muñoz-Couselo E., Soberino J., Racca F., Cortes J. (2018). Targeting FGFR pathway in breast cancer. Breast.

[49] André F., Cortés J. (2015). Rationale for targeting fibroblast growth factor receptor signaling in breast cancer. Breast Cancer Res. Treat..

[50] Adams B.D., Wali V.B., Cheng C.J., Inukai S., Booth C.J., Agarwal S., Rimm D.L., Győrffy B., Santarpia L., Pusztai L. (2016). miR-34a Silences c-SRC to Attenuate Tumor Growth in Triple-Negative Breast Cancer. Cancer Res..

[51] Tzeng Y.T., Liu P.F., Li J.Y., Liu L.F., Kuo S.Y., Hsieh C.W., Lee C.H., Wu C.H., Hsiao M., Chang H.T. (2018). Kinome-Wide siRNA Screening Identifies Src-Enhanced Resistance of Chemotherapeutic Drugs in Triple-Negative Breast Cancer Cells. Front. Pharmacol..

[52] Tryfonopoulos D., Walsh S., Collins D.M., Flanagan L., Quinn C., Corkery B., McDermott E.W., Evoy D., Pierce A., O’Donovan N. (2011). Src: A potential target for the treatment of triple-negative breast cancer. Ann. Oncol..

[53] Shen J., Li L., Howlett N.G., Cohen P.S., Sun G. (2020). Application of a Biphasic Mathematical Model of Cancer Cell Drug Response for Formulating Potent and Synergistic Targeted Drug Combinations to Triple Negative Breast Cancer Cells. Cancers.

[54] Shen J., Li L., Yang T., Cohen P.S., Sun G. (2020). Biphasic Mathematical Model of Cell-Drug Interaction That Separates Target-Specific and Off-Target Inhibition and Suggests Potent Targeted Drug Combinations for Multi-Driver Colorectal Cancer Cells. Cancers.

[55] Chou T.C. (2010). Drug combination studies and their synergy quantification using the Chou-Talalay method. Cancer Res..

[56] Rashband W.S. ImageJ 1.53K.

[57] Kupcho K., Shultz J., Hurst R., Hartnett J., Zhou W., Machleidt T., Grailer J., Worzella T., Riss T., Lazar D. (2019). A real-time, bioluminescent annexin V assay for the assessment of apoptosis. Apoptosis.

[58] Yang W., Soares J., Greninger P., Edelman E.J., Lightfoot H., Forbes S., Bindal N., Beare D., Smith J.A., Thompson I.R. (2013). Genomics of Drug Sensitivity in Cancer (GDSC): A resource for therapeutic biomarker discovery in cancer cells. Nucleic Acids Res..

[59] Langlois A.J., Holder W.D., Iglehart J.D., Nelson-Rees W.A., Wells S.A., Bolognesi D.P. (1979). Morphological and biochemical properties of a new human breast cancer cell line. Cancer Res..

[60] Riaz M., van Jaarsveld M.T., Hollestelle A., Prager-van der Smissen W.J., Heine A.A., Boersma A.W., Liu J., Helmijr J., Ozturk B., Smid M. (2013). miRNA expression profiling of 51 human breast cancer cell lines reveals subtype and driver mutation-specific miRNAs. Breast Cancer Res..

[61] Tate J.G., Bamford S., Jubb H.C., Sondka Z., Beare D.M., Bindal N., Boutselakis H., Cole C.G., Creatore C., Dawson E. (2019). COSMIC: The Catalogue Of Somatic Mutations In Cancer. Nucleic Acids Res..

[62] Thota R., Johnson D.B., Sosman J.A. (2015). Trametinib in the treatment of melanoma. Expert Opin. Biol. Ther..

[63] Yoshida T., Kakegawa J., Yamaguchi T., Hantani Y., Okajima N., Sakai T., Watanabe Y., Nakamura M. (2012). Identification and characterization of a novel chemotype MEK inhibitor able to alter the phosphorylation state of MEK1/2. Oncotarget.

[64] Khan Z.M., Real A.M., Marsiglia W.M., Chow A., Duffy M.E., Yerabolu J.R., Scopton A.P., Dar A.C. (2020). Structural basis for the action of the drug trametinib at KSR-bound MEK. Nature.

[65] Gonzalez-Del Pino G.L., Li K., Park E., Schmoker A.M., Ha B.H., Eck M.J. (2021). Allosteric MEK inhibitors act on BRAF/MEK complexes to block MEK activation. Proc. Natl. Acad. Sci. USA.

[66] Maiello M.R., D’Alessio A., Bevilacqua S., Gallo M., Normanno N., De Luca A. (2015). EGFR and MEK Blockade in Triple Negative Breast Cancer Cells. J. Cell Biochem..

[67] Zhou Y., Lin S., Tseng K.F., Han K., Wang Y., Gan Z.H., Min D.L., Hu H.Y. (2016). Selumetinib suppresses cell proliferation, migration and trigger apoptosis, G1 arrest in triple-negative breast cancer cells. BMC Cancer.

[68] Chou T.C., Talalay P. (1984). Quantitative analysis of dose-effect relationships: The combined effects of multiple drugs or enzyme inhibitors. Adv. Enzym. Regul..

[69] Pircher M., Winder T., Trojan A. (2021). Response to Vemurafenib in Metastatic Triple-Negative Breast Cancer Harbouring a BRAF V600E Mutation: A Case Report and Electronically Captured Patient-Reported Outcome. Case Rep. Oncol..

[70] Wang L., Lu Q., Jiang K., Hong R., Wang S., Xu F. (2022). BRAF V600E Mutation in Triple-Negative Breast Cancer: A Case Report and Literature Review. Oncol. Res. Treat..

[71] Seo T., Noguchi E., Yoshida M., Mori T., Tanioka M., Sudo K., Shimomura A., Yonemori K., Fujiwara Y., Tamura K. (2020). Response to Dabrafenib and Trametinib of a Patient with Metaplastic Breast Carcinoma Harboring a BRAF V600E Mutation. Case Rep. Oncol. Med..

[72] Madsen R.R., Knox R.G., Pearce W., Lopez S., Mahler-Araujo B., McGranahan N., Vanhaesebroeck B., Semple R.K. (2019). Oncogenic PIK3CA promotes cellular stemness in an allele dose-dependent manner. Proc. Natl. Acad. Sci. USA.

[73] Nijman S.M. (2011). Synthetic lethality: General principles, utility and detection using genetic screens in human cells. FEBS Lett..

[74] Topatana W., Juengpanich S., Li S., Cao J., Hu J., Lee J., Suliyanto K., Ma D., Zhang B., Chen M. (2020). Advances in synthetic lethality for cancer therapy: Cellular mechanism and clinical translation. J. Hematol. Oncol..

[75] Lord C.J., Ashworth A. (2017). PARP inhibitors: Synthetic lethality in the clinic. Science.

