# Supplementary material for: Identification of Lethal Inhibitors and Inhibitor Combinations for Mono-Driver versus Multi-Driver Triple-Negative Breast Cancer Cells
Source: Cancers (Basel). 2022 Aug 20;14(16):4027. doi: 10.3390/cancers14164027 (PMC9407008; doi:10.3390/cancers14164027)
Supplement: Supplementary file 1 [file cancers-14-04027-s001.zip › Revised Supporting information-Full images of Western blots.pptx]

## Slide 1
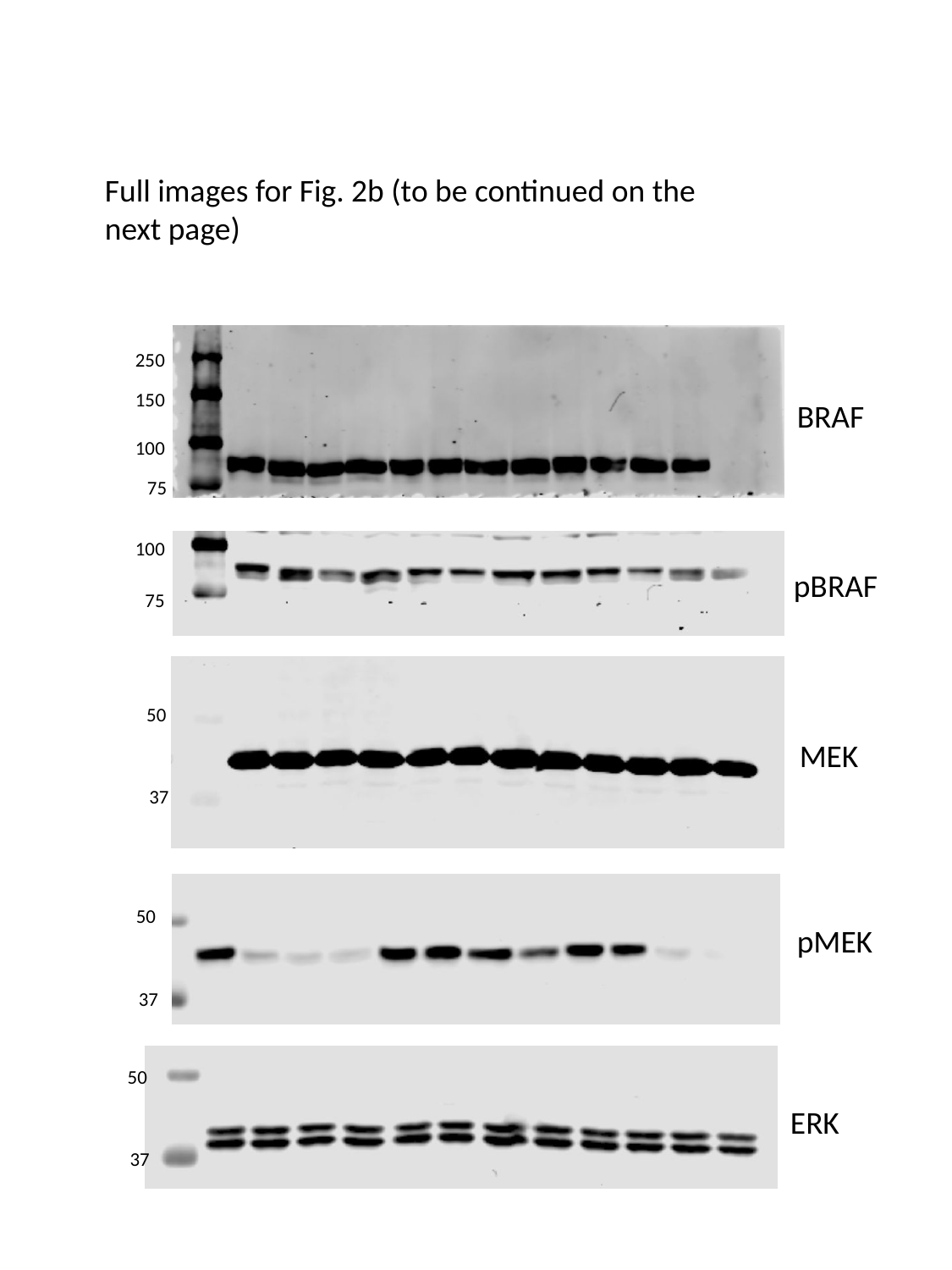

Full images for Fig. 2b (to be continued on the next page)
250
150
BRAF
100
75
100
pBRAF
75
50
MEK
37
50
pMEK
37
50
ERK
37

## Slide 2
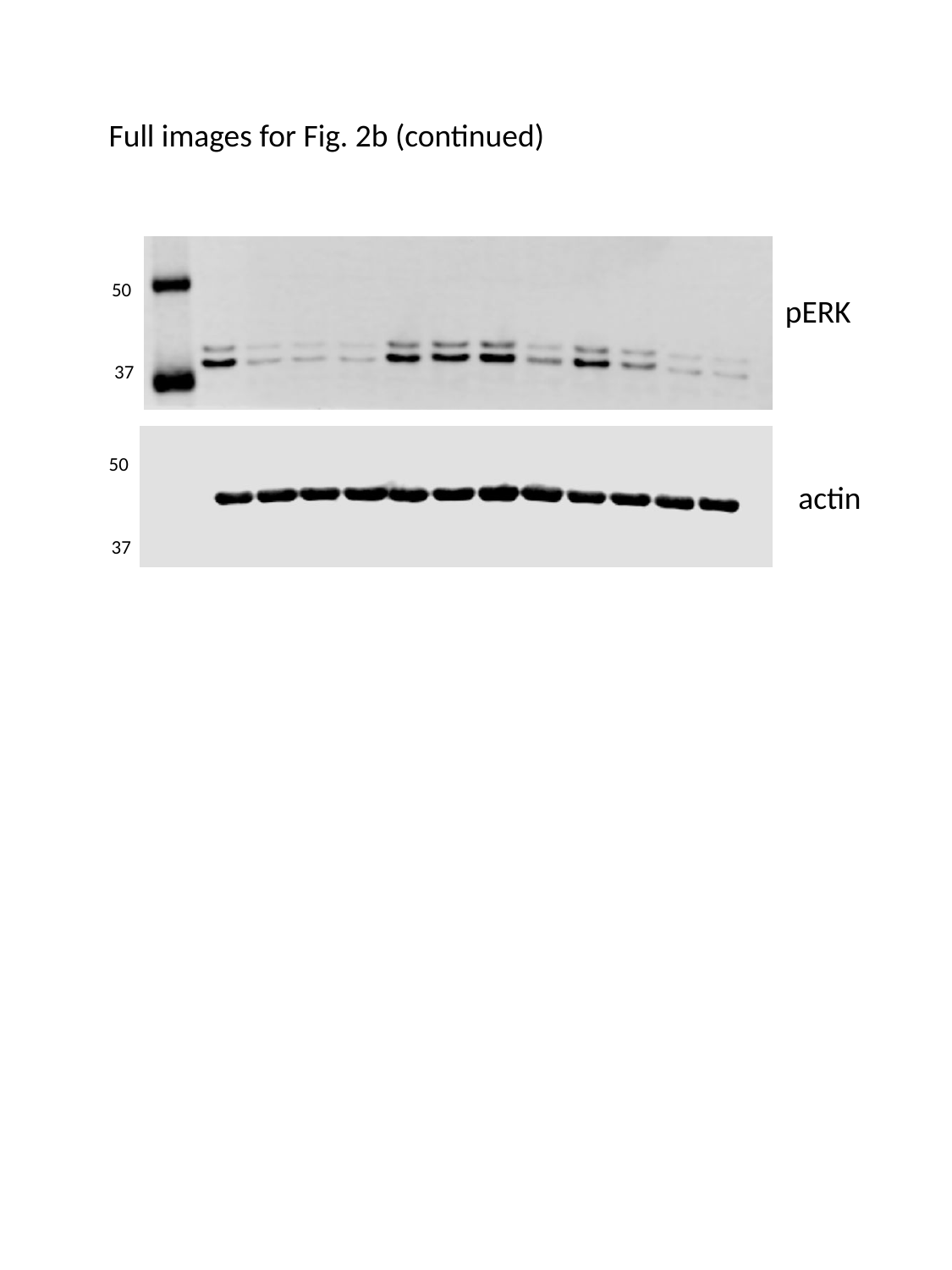

Full images for Fig. 2b (continued)
50
pERK
37
50
actin
37

## Slide 3
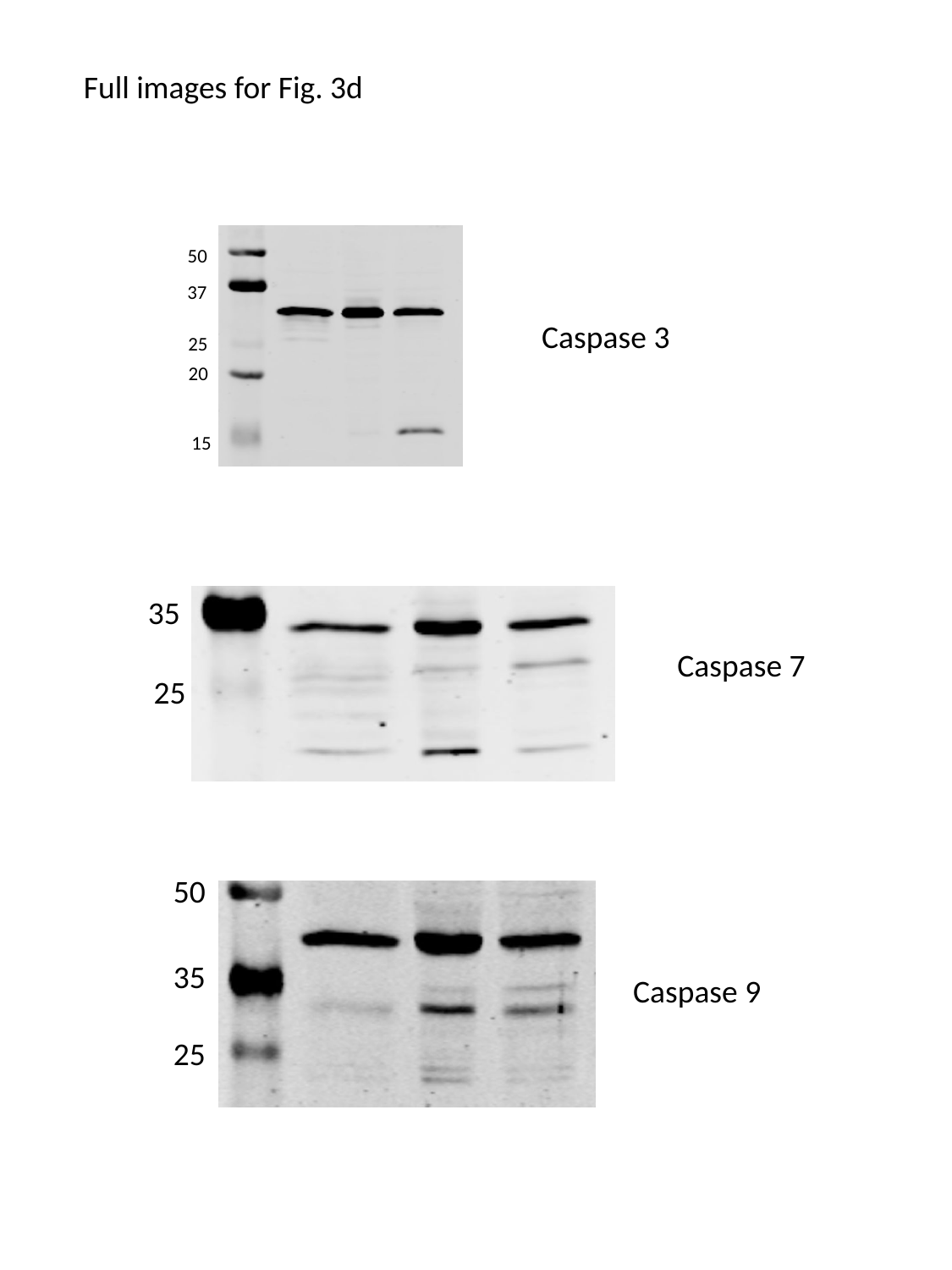

Full images for Fig. 3d
50
37
Caspase 3
25
20
15
35
Caspase 7
25
50
35
Caspase 9
25

## Slide 4
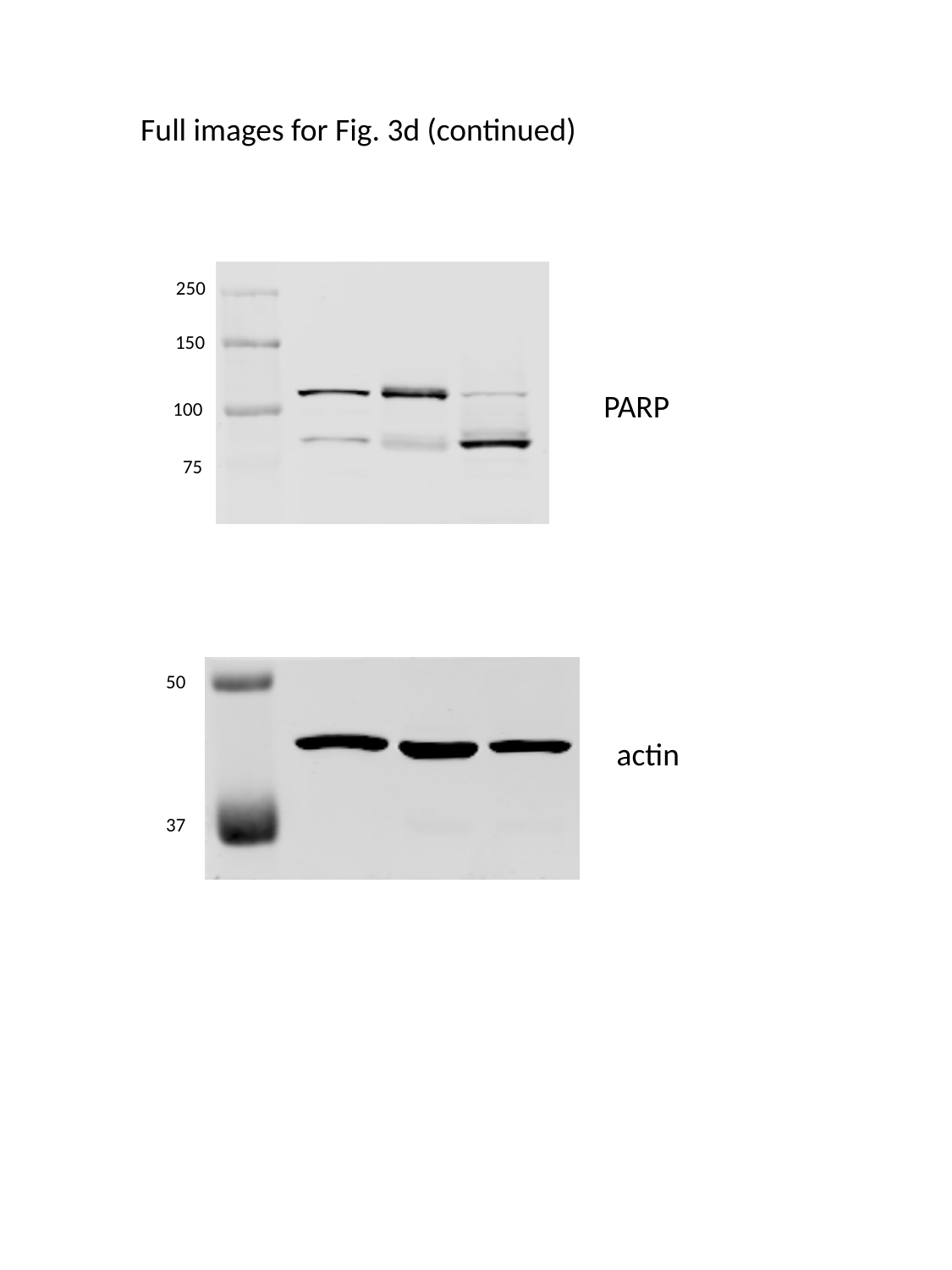

Full images for Fig. 3d (continued)
250
150
PARP
100
75
50
actin
37

## Slide 5
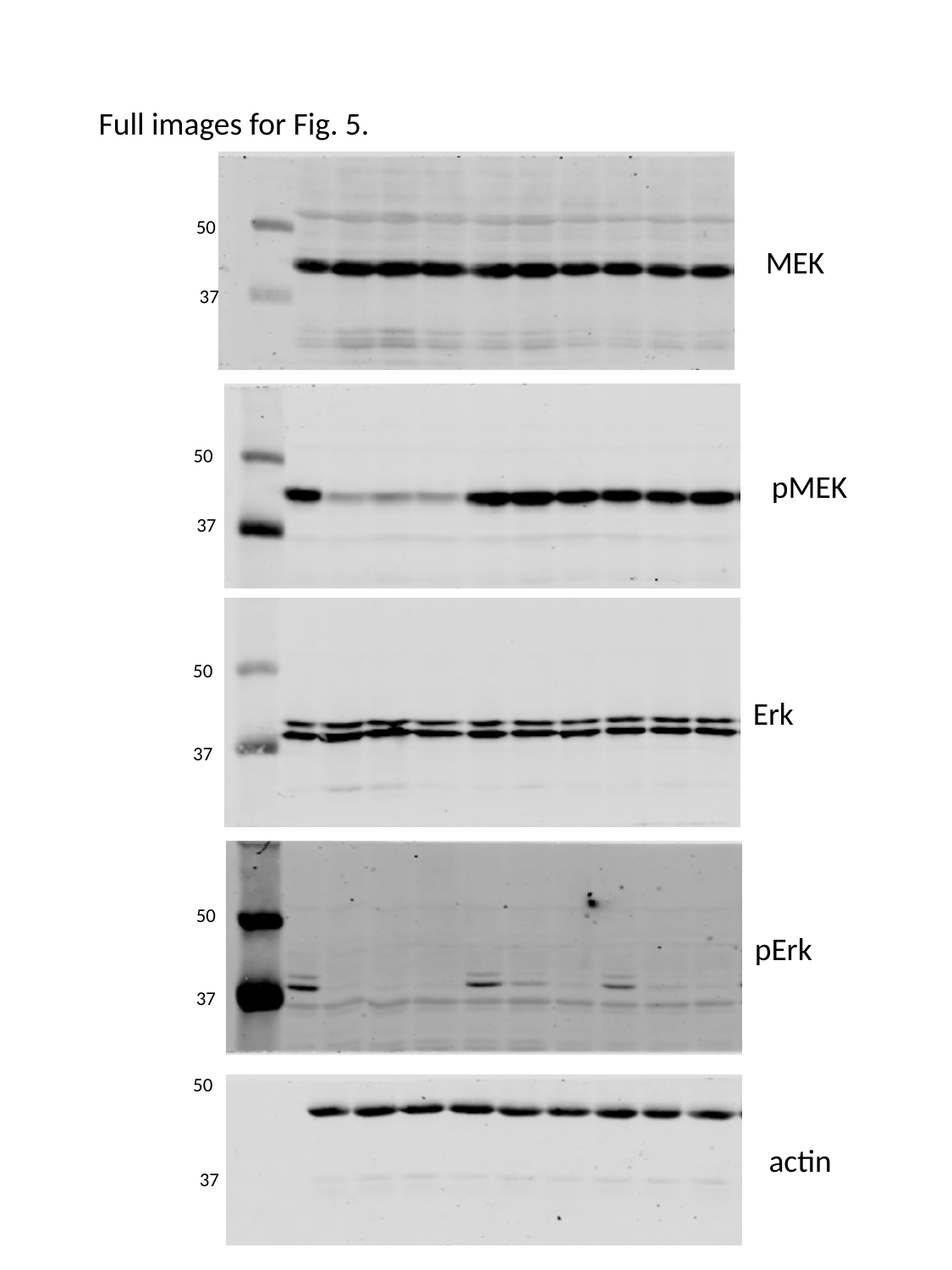

Full images for Fig. 5.
50
MEK
37
50
pMEK
37
50
Erk
37
50
pErk
37
50
actin
37

## Slide 6
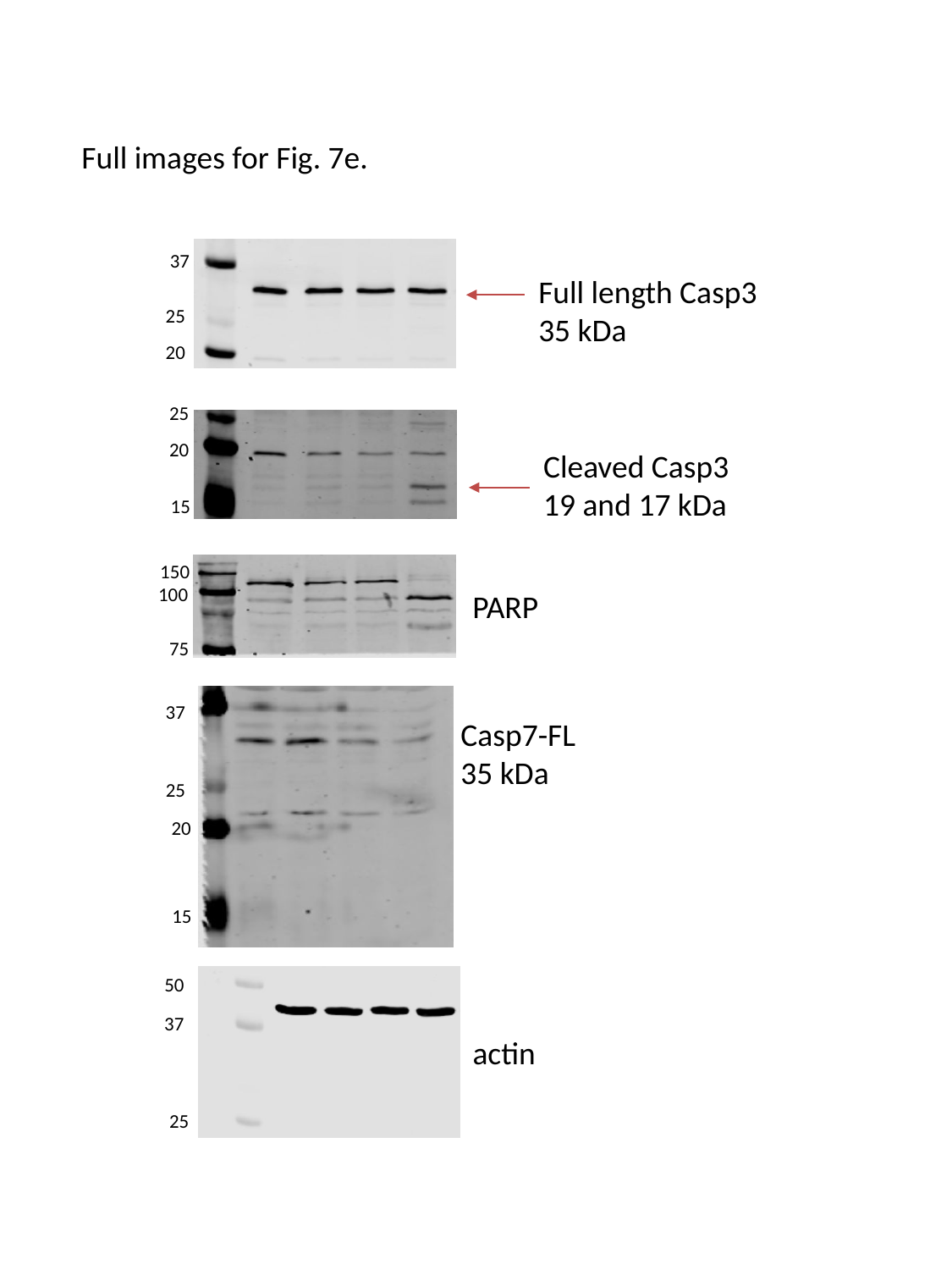

Full images for Fig. 7e.
37
Full length Casp3
35 kDa
25
20
25
20
Cleaved Casp3
19 and 17 kDa
15
150
100
PARP
75
37
Casp7-FL
35 kDa
25
20
15
50
37
actin
25
